# Factors associated with measles vaccination status in children under the age of three years in a post-soviet context: a cross-sectional study using the DHS VII in Armenia

**DOI:** 10.1186/s12889-021-10583-5

**Published:** 2021-03-20

**Authors:** Annabell C. Kantner, Sibylle Herzig van Wees, Erik M. G. Olsson, Shirin Ziaei

**Affiliations:** 1 Department of Women’s and Children’s Health, Uppsala University, Akademiska sjukhuset, SE-751 85 Uppsala, Sweden; 2 Karolinska Institutet, Department of Global Public Health, K9 Global folkhälsa, K9 GH Stålsby Lundborg Hanson, SE-171 77, Stockholm Sweden; 3 Clinical Psychology in Healthcare, Department of Women’s and Children’s Health, Uppsala University, Akademiska sjukhuset, SE-751 85 Uppsala, Sweden

**Keywords:** Measles vaccination, MMR, Children, Armenia

## Abstract

**Background:**

The resurgence of measles globally and the increasing number of unvaccinated clusters call for studies exploring factors that influence measles vaccination uptake. Armenia is a middle-income post-Soviet country with an officially high vaccination coverage. However, concerns about vaccine safety are common. The purpose of this study was to measure the prevalence of measles vaccination coverage in children under three years of age and to identify factors that are associated with measles vaccination in Armenia by using nationally representative data.

**Methods:**

Cross-sectional analysis using self-report data from the most recent Armenian Demographic Health Survey (ADHS VII 2015/16) was conducted. Among 588 eligible women with a last-born child aged 12–35 months, 63 women were excluded due to unknown status of measles vaccination, resulting in 525 women included in the final analyses. We used logistic regression models in order to identify factors associated with vaccination status in the final sample. Complex sample analyses were used to account for the study design.

**Results:**

In the studied population 79.6% of the children were vaccinated against measles. After adjusting for potential confounders, regression models showed that the increasing age of the child (AOR 1.07, 95% CI: 1.03–1.12), secondary education of the mothers (AOR 3.38, 95% CI: 1.17–9.76) and attendance at postnatal check-up within two months after birth (AOR 2.71, 95% CI: 1.17–6.30) were significantly associated with the vaccination status of the child.

**Conclusions:**

The measles vaccination coverage among the children was lower than the recommended percentage. The study confirmed the importance of maternal education and attending postnatal care visits. However, the study also showed that there might be potential risks for future measles outbreaks because of delayed vaccinations and a large group of children with an unknown vaccination status.

## Background

Despite the fact that vaccinations are one of the most successful and cost-effective public health interventions to reduce mortality and morbidity, approximately 1.4 million children die from vaccine-preventable diseases globally every year [1–3]. Improvements in vaccination coverage during recent decades have led to rapid reduction in rates of vaccine-preventable diseases, but inequalities in vaccination coverage persist [4, 5].

Among those vaccine preventable diseases, measles is one of the most contagious [6]. Measles vaccination is often given in two doses of a combination vaccine (Measles, Mumps, Rubella (MMR)) and is considered to be safe and effective [7]. In order to reach community-level immunity and eliminate measles, a vaccination coverage level of 95% with two doses is needed [8–10]. Studies have also shown that the measles-containing vaccine (MCV) not only prevent measles infection but also is associated with a reduced risk of all-cause mortality [5, 11].

After a long period of stability, large measles outbreaks occurred in several World Health Organization (WHO) regions [12] with more than 140,000 deaths worldwide in 2018 [13]. These outbreaks have been attributed to insufficient vaccination rates in some settings [14] or unvaccinated clusters, which occur even in countries with high vaccination rates [15]. These unvaccinated clusters make measles a persisting public health threat especially in a globalized world with increasing traveling habits [16].

Unvaccinated clusters often consist of individuals with vaccine hesitant attitudes within expanding antivaccination networks [15]. During the past decade, research has shown that people both in high- and low-income countries have lost confidence in some vaccines and particularly in the measles vaccine [17, 18]. Especially the Wakefield scandal, which falsely associated MMR vaccination with autism [19] contributed to a decreased confidence in the measles vaccination.

In order to increase vaccination rates and to target interventions in unvaccinated clusters, there is a need to understand the factors associated to vaccination uptake [20, 21]. Previous studies have identified a variety of factors that potentially influence measles vaccination. For example the *maternal level of education* [2, 4, 5, 22–24], *marital status* [2, 25]*,* the socioeconomic status such as *wealth index* [1, 4, 23, 26, 27] and exposure to *mass media* [15, 28] were identified. However, those studies were mainly done in low-income countries and a few in high-income countries with differing results depending on the setting. Research on factors influencing measles vaccination from middle-income countries, including Armenia, is scarce.

Armenia is a post-Soviet upper-middle income country [29], which underwent the transition from a socialist to a market-style economy at severe socioeconomic cost [30]. Access to primary care is limited due to a strongly centralized health care system and high out of pocket expenditures for health care [31].

Armenia follows WHO guidelines regarding childhood vaccination which includes Bacillus Calmette–Guérin (BCG) vaccine against tuberculosis administered at birth, three doses of a diphtheria, pertussis and tetanus vaccine (DTP) and three doses of a polio vaccine (Pol) both administered at 6, 12 and 18 weeks and a measles-containing vaccine (MCV) administered at the age of one year [32]. Officially, the immunization coverage for key vaccine-preventable diseases is high in Armenia, although often shortly beneath rates that are required to reach herd immunity. The mean percentage of measles vaccination coverage in 2010–2015 with two doses MMR was 94.3 [8]. However, there appears to be a concern over vaccine safety in the region [17, 33] and 19 measles cases were reported in 2018 in the country. Further there have been measles outbreaks reported in countries from the geographical region, such as in Kazakhstan in 2015–2016 with 2341 measles cases where the majority was either not vaccinated or had no reliable documentation on their vaccination status [6]. Such outbreaks might increase the risk that measles spread to Armenia and thus highlights the importance of maintaining high vaccination coverage in the country.

In order to increase and maintain a high vaccination coverage, identifying factors which are associated with vaccination uptake is necessary. To the best of our knowledge, studies evaluating such factors are scarce in the context of post-soviet countries. Therefore, the purpose of this study is to fill the knowledge gap by measuring the prevalence of measles vaccination coverage in children under three years of age and to identify demand side factors that are associated with measles vaccination in the post-Soviet context of Armenia by using nationally representative data.

## Methods

This cross-sectional analysis uses self-report data from the most recent version of the Armenian Demographic and Health Survey 2015/16 (ADHS). The ADHS is a nationally representative household survey that provides data for a wide range of indicators, including reliable information about vaccination coverage, which is independent from the official health reports [34, 35].

**Fig. 1:**
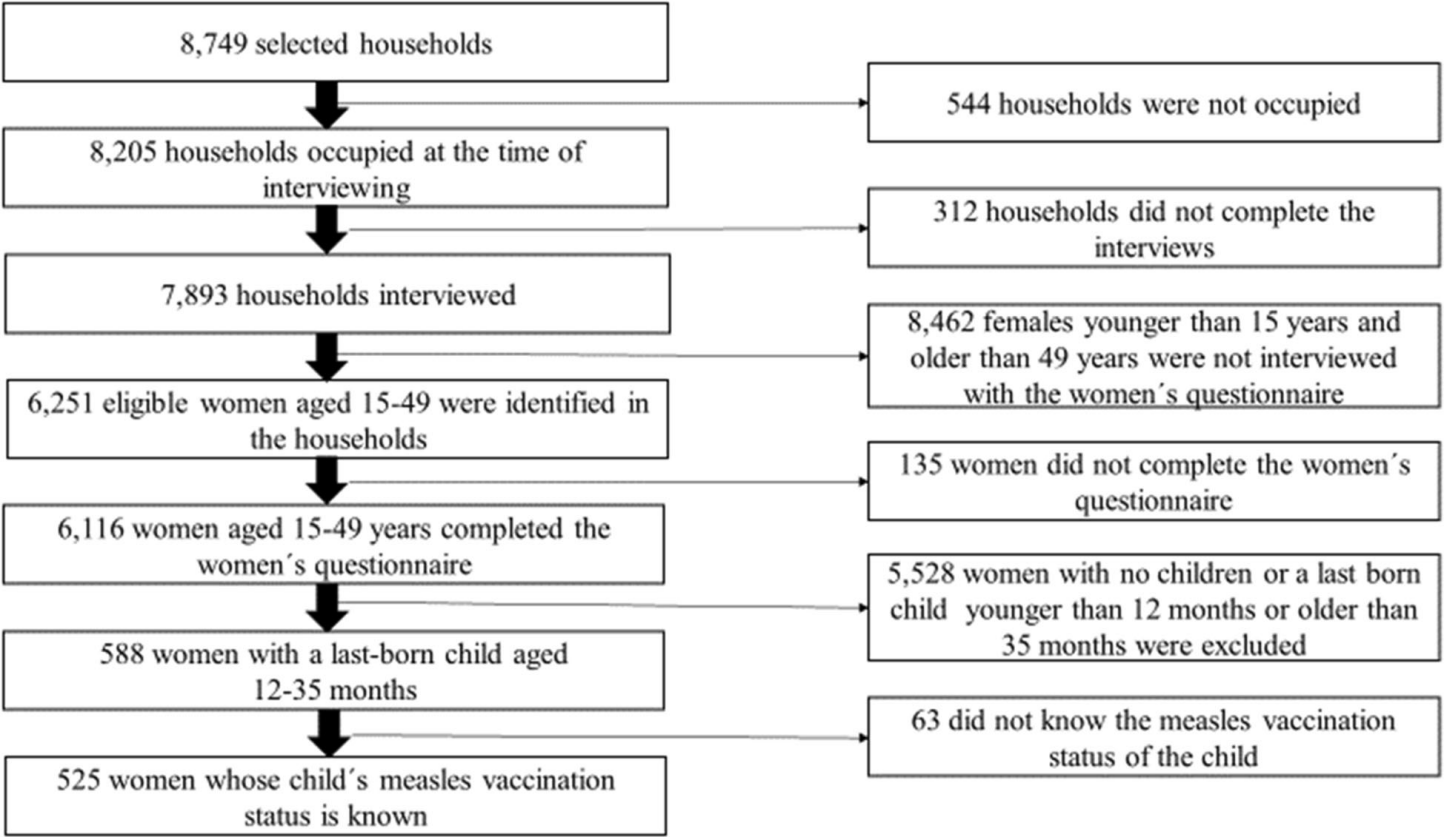
Selection process of the eligible women aged 15–49 years old using the Armenian Demographic Health Survey 2015/16

The sampling technique used by ADHS is multi-staged. Based on the sampling frame of the Armenian Population and Housing Census from 2011, 11,571 Enumeration areas (EAs) covering the whole country were created. Out of these, a representative probability sample of 8749 households were selected, and 7893 households were successfully interviewed. Women were considered eligible for the survey if they were between 15 and 49 years old and if they were permanent resident of the household or spent the night before the survey in the household. A total of 6116 eligible women completed the women’s questionnaire interview. In the ADHS, the vaccination status was recorded for children below the age of 36 months for all vaccinations [36]. According to the Armenian vaccination schedule, children should receive the first dose of measles vaccination at the age of 12 months [32]. Therefore, women with a last-born child in the age between 12 and 35 months were selected for the current study (*n* = 588). We excluded 63 participants because they were not aware of the measles vaccination status of the child. The final study population consisted of 525 mothers and their last-born children at the age of 12–35 months (see Fig. 1).

### Independent variables

Based on the results from previous studies [1, 2, 4, 23] and the Armenian context [30, 34, 37], a variety of factors have been selected as independent variables. As general factors, we selected residence in urban or rural areas and the wealth index, which was a composition of household assets provided in quintiles. The chosen maternal factors were age in years, the marital status, measuring whether the women were either currently or formerly in union, and the level of education. For the maternal level of education, we used three levels of education: basic (grades 1–9), secondary (grades 10–12) and higher education (secondary special, which provides training for careers that require mid-level qualifications such as nurses or technicians, and university education) [36].

As paternal factors we used the age of the partner in years and the level of education [38]. The partners´ levels of education was divided in three categories: none or primary, secondary, and secondary special. Child related factors included in the study were the age of the child in months, gender, and the birth order, categorized into first-born, second-born and third-or higher born.

We also looked at factors representing health seeking behaviour, such as whether the distance to the health facility was perceived as a big problem or not, the number of antenatal care visits and attendance to a postnatal care visit two months after birth (yes, no).

To elaborate the influence of mass media, we combined three variables, including watching TV, listening to the radio, and reading the newspaper. The women were asked how often they have used the listed media and their responses ranged from “not at all=0”, “less than once a week=1” to “at least once a week=2”. The answers to all three questions were summarized and was then categorized into low exposure (total score 0–2) and high exposure (total score equal or above 3).

Information about the measles vaccination as the outcome variable was obtained based on mothers recall or from the child health card with or without a date. The variable was categorized as “vaccinated” if the child received MMR and “not vaccinated” otherwise.

### Statistical analysis

Complex sample analyses were performed in order to account for cluster sampling design and sample weight in DHS data [39]. Descriptive characteristics of women and their children were presented with frequency (percentage) for categorical and mean (Standard Deviation (SD)) for continuous variables. We used chi-square tests for categorical and Student’s *t*-Test or one-way ANOVAs for continuous variables in order to evaluate bivariate associations between children’s background characteristics and their vaccination status. Factors associated with the outcome of interest with a *p-*value < 0.2 were included in the multivariate analyses. We used logistic regression models in order to identify factors associated with vaccination status. The results of this paper are presented with crude OR, where each exposure variable is analysed separately with the outcome of interest. In Model 1, factors associated with the outcome of interest (*p* < 0.2) in bivariate analyses were included. Model 2 additionally included wealth index and place of residence as they have been suggested as important predictors of child vaccination status in previous research [1, 4, 20, 23]. *P* values < 0.05 has been considered significant in the models. Statistical software package IBM SPSS Statistics version 24 (IBM, SPSS, Armonk,NY, USA) was used to perform the analysis.

### Ethical considerations

The analysis of this study was based on existing survey data collected by the DHS (The DHS Programme, www.dhsprogram.com). The survey was approved by the ICF Institutional Review Board. The DHS has strict rules on providing informed consent to participants and ensure that the participants identity is protected. Before accessing the DHS data from the webpage an electronic registration form highlighting the desired data and plan for analysis was submitted and approved by the DHS.

## Results

### Characteristics of the participants

A total of 525 women and their last-born child in the age of 12 to 35 months were included in the study (see Fig. 1). The background characteristics of the mothers and their children based on vaccination status are presented in Table 1. The mean age of the mothers was 28.35 years, 56.9% live in urban areas and 26.8% belonged to the richest wealth index quintile. More than half of the mothers had higher education (53.8%), around 97% were currently in a relationship and the mean age of their partners was 32.54 years. Among the partners, almost 10% had none or primary education, the rest had secondary or secondary special education.

**Table 1:** Background characteristics of mothers and the measles vaccinations status of their last-born child aged 12–35 months, using the Armenian demographic and health survey 2015/16 (*n* = 525)

	Overall frequency (%)/ Mean (SD) (***n*** = 525)	missing	Not received MMR (***n*** = 107) Frequency (%)/ Mean (SD)	Received MMR (***n*** = 418) Frequency (%)/ Mean (SD)	***p***-value^**1**^
***Maternal age (years)***					
	28.35 (4.83)		28.32 (4.94)	28.36 (0.48)	0.96
***Type of place of residence***					
Urban	299 (56.9)		65 (60.7)	234 (56)	0.43
Rural	226 (43.1)		42 (39.3)	184 (44)	
***Wealth index quintiles***					
Poorest	108 (20.5)		17 (15.9)	90 (21.6)	0.66
Poorer	100 (19.0)		20 (18.7)	80 (19.2)	
Middle	96 (18.2)		18 (16.8)	78 (18.7)	
Richer	81 (15.4)		20 (18.7)	60 (14.4)	
Richest	141 (26.8)		32 (29.9)	109 (26.1)	
***Maternal educational level***					
Basic	35 (6.6)		12 (11.3)	23 (5.5)	0.06
Secondary	207 (39.5)		32 (30.2)	175 (41.9)	
Higher	283 (53.8)		62 (58.5)	220 (52.6)	
***Relationship status***					
Currently in a relationship	511 (97.3)		104 (97.2)	407 (97.4)	0.88
Formerly in a relationship	14 (2.7)		3 (2.8)	11 (2.6)	
***Husband/partner’s age (years)***					
	32.54 (5.85)	14	32.13 (6.33)	32.65 (5.73)	0.42
***Husband/partner’s education level***					
None and Primary	51 (9.6)		11 (10.6)	140 (9.8)	0.84
Secondary	246 (46.9)		47 (45.2)	199 (48.9)	
Secondary special	214 (40.8)		46 (42.4)	168 (41.3)	
		14			
***Distance to the health facility***					
Big problem	54 (10.2)		11 (10.4)	42 (10)	0.87
Not a big problem	471 (89.8)		95 (89.6)	376 (90)	
***Number of antenatal care visits***					
	7.79 (3.67)	7	7.37 (2.69)	7.90 (3.87)	**0.04**
***Postnatal visit within 2 months after birth***					
No	32 (6.2)		13 (12.1)	20 (4.8)	**0.01**
Yes	492 (93.8)		94 (87.9)	398 (95.2)	
***Level of mass media exposure***					
Low	263 (50.2)		61 (57.0)	203 (48.6)	0.15
High	261 (49.7)		46 (43.0)	215 (51.4)	
***Child’s age (months)***					
	22.57 (6.74)		20.29 (7.19)	23.15 (6.49)	**0.03**
***Sex of the child***					
Male	269 (51.3)		56 (52.3)	214 (51.2)	0.88
Female	256 (48.7)		51 (47.7)	204 (48.8)	
***Birth order***					
First	190 (36.3)		44 (41.1)	146 (34.9)	0.5
Second	211 (40.1)		42 (39.3)	169 (40.4)	
Third and more	124 (23.6)		21 (19.6)	103 (24.6)	
***Measles vaccination status***					
Vaccinated	418 (79.6)				
Not	107 (20.4)				

*SD* Standard deviation, *MMR* Measles Mumps and Rubella vaccine

^1^ comparison between the vaccinated and unvaccinated group by using chi-square or Student’s *t-*test/one-way ANOVAs

The majority of the women (89.4%) did not perceive the distance to the health facility as a problem. The women received on average 7.79 antenatal care visits and almost 94% attended postnatal care visits two months after birth. Media exposure was low in approximately half of the women.

The mean age of the children was 22.6 months and 51.3% were boys. The children were mostly first and second born and 79.6% were vaccinated against measles. Among vaccinated children, vaccination status of 51% were recorded on the health cards and rest were based on mothers’ recall. The mean age of children who were vaccinated against measles was 23.15 months and only 2.9% (12 out of 418) children were vaccinated against measles at the age of 12 months (data not shown).

Children were more likely to be vaccinated against measles if they were older (*p* = 0.03), if they had a postnatal care visit within two months after birth (*p* = 0.01) and if their mothers had attended more antenatal care visits (*p* = 0.04) (Table 1).

In the crude logistic regressions, the age of the child, the maternal education level and attending postnatal care visits within two months after birth were significantly associated with the vaccination status of the child. The odds of being vaccinated were increasing with the increasing age of the child (OR 1.07; 95% CI: 1.02–1.12). Compared to children of mothers with basic education, children of mothers with secondary education were significantly more likely to be vaccinated (OR 2.86, 95% CI: 1.09–7.56). Also, children of mothers with higher education were more likely to be vaccinated, but the result was not significant (OR 1.87, 95% CI: 0.74–4.74). Furthermore, attending postnatal care visits within two months after birth increased the likelihood of the child being vaccinated more than twofold (OR 2.65, 95% CI: 1.29–5.45) (Table 2).

**Table 2:** Results of the binary and multiple logistic regression models to estimate the odds of being vaccinated against measles in children aged 12–35 months, using Armenian demographic and health survey 2015/16 (*n* = 525)

	Crude OR (95%CI)	Model 1 AOR^**1**^ (95%CI)	Model 2 AOR^**2**^ (95%CI)
***Maternal education***			
Basic education	Ref	Ref	Ref
Secondary education	**2.86 (1.09–7.56)**	**2.76 (1.00–7.62)**	**3.38 (1.17–9.76)**
Higher education	1.87 (0.74–4.74)	1.52 (0.58–4.02)	2.24 (0.78–6.40)
***Number of antenatal care visits***			
	1.05 (0.98–1.11)	1.05 (0.98–1.12)	1.05 (0.98–1.12)
***Postnatal checks within 2 months***			
No	Ref	Ref	Ref
Yes	**2.65 (1.29–5.45)**	**2.53 (1.14–5.63)**	**2.71 (1.17–6.30)**
***Media exposure***			
Low media exposure	Ref	Ref	Ref
High media exposure	1.40 (0.88–2.20)	1.37 (0.85–2.20)	1.47 (0.92–2.34)
***Child’s age (months)***			
	**1.07 (1.02–1.12)**	**1.07 (1.03–1.12)**	**1.07 (1.03–1.12)**
***Type of place of residence***			
Rural	Ref	–	Ref
Urban	0.82 (0.50–1.34)	–	0.99 (0.50–1.95)
***Wealth index quintile***			
Poorest	Ref	–	Ref
Poorer	0.79 (0.37–1.69)	–	0.67 (0.31–1.53)
Middle	0.85 (0.37–1.96)	–	0.67 (0.26–1.73)
Richer	0.58 (0.25–1.30)	–	0.42 (0.14–1.28)
Richest	0.65 (0.30–1.45)	–	0.48 (0.18–1.30)

*OR* Odds Ratio, *AOR* Adjusted Odds Ratio, *CI* Confidence interval

^1^ Model adjusting for maternal education, number of antenatal care visits, postnatal checks, media exposure and current age of the child

^2^ Model adjusting for maternal education, number of antenatal care visits, postnatal checks, media exposure, current age of the child, the type of residence and wealth index quintile

When adjusting for the potential confounders in Model 1, the odds of being vaccinated remained significantly associated with the children’s age, maternal education and attending a postnatal visit two months after birth. Children were more likely to be vaccinated if they were older (AOR 1.07, 95% CI: 1.03–1.12). Mothers who had secondary education were significantly more likely to vaccinate their children compared to those with basic education (AOR 2.76, 95% CI: 1.00–7.62). Attending postnatal care visits within two months after birth was also associated with higher odds of the child being vaccinated against measles (AOR 2.53, 95% CI: 1.14–5.63) (Table 2).

Additionally adjusting for place of residence and wealth index in Model 2 did not have an impact on the significance of the results. The current age of the child (AOR 1.07; 95% CI: 1.03–1.12), secondary level of education in mothers (AOR 3.38; 95% CI: 1.17–9.76) and attending postnatal visits within two months after birth (AOR 2.71; 95% CI: 1.17–6.30) stayed significantly associated with the vaccination status of the child. The latter two with even larger odds (Table 2).

## Discussion

The aim of this study was to measure the measles vaccination coverage and to identify factors that were associated with measles vaccination status among children under 3 years of age in Armenia. The analysis showed that the vaccination coverage is lower than the recommended coverage with only 79.6% of the 12–35 months old children being vaccinated with the first dose of MMR. We also have identified that children were more likely to be vaccinated if they were older and if their mothers had secondary education and attended postnatal care visits two months after birth.

### Vaccination coverage

Our study showed that the vaccination coverage in the studied population is far below the recommended 95% coverage and below the officially reported coverage [8]. The discrepancy between household surveys and reports from WHO/UNICEF has been recognized in previous studies [37] because they are largely based on administrative data from health-service-provider registries with potential weak administrative data systems [40]. There is a risk of over-reporting in these systems [40] and this may indicate that the actual coverage might be lower than official reports.

### Possible delay in vaccination

In the studied population, the mean age of the children in the vaccinated group was 23.15 months. Further a positive association between the age of the child and the probability of being vaccinated was observed. In both logistic regression models, the odds of being vaccinated increased with the increasing age of the child.

According to the Armenian vaccination schedule, measles vaccination should be given at the age of 12 months. Vaccinations given later than four weeks after the recommended age are considered to be not given on time [34] indicating that many children in the studied population may be vaccinated too late. Such delays in vaccination allow for longer periods of susceptibility [41] and could increase the possibility of measles transmissions in the society [26]. Previous studies have shown that incorrectly timed vaccinations were associated with outbreaks and re-emergence of a vaccine preventable disease [41].

The problem of delayed vaccinations is already known in Armenia. Schweizer et al. [34] showed that there have been improvements in the timeliness administration of the measles vaccine from year 2000 to 2010 in Armenia. However, our study indicated that there is still a potential delay. Agopian et al. [41] came to same conclusion and showed that the administration of Measles containing vaccines in Armenia was delayed with a median age of 61.1 weeks by analysing the ADHS 2015/2016.

### Association between maternal level of education and measles vaccination of the child

The maternal level of education was associated with the vaccination status in this study. We found mothers with secondary level of education were more likely to vaccinate their child against measles than those with basic education. This is in line with the results from previous studies, where better education in mothers was associated with better immunization coverage [1, 20, 23, 24]. Often this is explained by the fact that educated mothers are more likely to remember dates, understand the importance of vaccination [22] or may have a higher income and thereby able to pay fees.

Children of mothers with higher education were not significantly more likely to be vaccinated compared to mothers with basic education. This might be surprising considering the benefit of maternal education on the child’s health. However, this finding might show a trend in Armenia, which has been shown in other, mainly high-income settings where clusters of vaccine hesitancy may appear in highly educated groups [42]. Other studies have shown there is a tendency among better educated mothers to skip vaccinations [30] and that the highest level of education is not associated with more positive views on vaccine importance and effectiveness in Armenia [18].

However, children to mothers with basic education were less likely to be vaccinated against measles than children to better educated mothers. The results suggested that interventions should focus on mothers with lower education in order to increase measles vaccination coverage.

### Association between postnatal care visits and measles vaccination of the child

Postnatal care visits two months after birth were significantly associated with measles vaccination in all regression models. This association has only been shown in other studies from the African context [25]. Attending postnatal care visits may represent health seeking behaviour and women who seek to see a health care professional for a check-up might be more likely to seek care for their child later. Furthermore, additional information about vaccination might have been provided during that visit and a positive relationship to the health care system might have been established [27].

These results show that attending postnatal care visits might be positively associated with the vaccination coverage and future interventions may benefit from encouraging mothers to attend postnatal care visits.

### The amount of don’t knows indicates a serious risk

Measles is a disease with a high communicability, and therefore high immunization rates and vaccine efficacy is needed for reaching herd immunity [7]. In the primary studied population, 63 mothers (11%) were not aware of the vaccination status of their child and had to be excluded. There is a chance that some of these children were vaccinated and the mother only did not remember whether one of the injections, the child received, was a measles containing vaccine. However, data from WHO shows that a high percentage of measles cases in Armenia have an unknown or unclear vaccination status [43]. Thus, it is likely that children with an unknown vaccination status in this study were not vaccinated, which poses a risk for an outbreak. Furthermore, children who are not vaccinated against measles do not benefit from the potential immune training effects on the overall mortality by the measles vaccine, which has been shown in previous studies [5, 11].

#### Strengths and weaknesses

Previous studies about factors associated with measles vaccinations were done in low- or high-income countries. This study adds knowledge about factors associated with measles vaccination in a middle- income post-soviet setting of Armenia. Furthermore, we have used the data collected by DHS, which is nationally representative and well described.

#### Limitations

The use of secondary data limited the inclusion of additional variables that could explain the measles vaccination status, for example the availability of immunization services, costs, attitudes and beliefs towards immunization or religion.

Additionally, the sample size was relatively small to show strong significant associations. Only mothers with a child younger than 36 months were asked for the immunization status of the child, which reduces the potential inclusion into this study population. Furthermore, the use of basic education as a reference category with a small sample of only 35 women limits the statistical power and may have led to an overestimation.

There is a risk of recall bias because information was obtained from mothers´ self-report. In general, maternal recall of vaccination status is considered to be reliable [20, 44], however, the risk of over or underreporting still remains. Further, the cross-sectional design of the study limits the ability to draw causal conclusions.

## Conclusion

This study suggested that in Armenia measles vaccination coverage among the children under the age of three might be less than the recommended coverage. The study shows that attending postnatal care visits two months after birth and the level of maternal education are associated with measles vaccination status of the children. However, the mean age of the vaccinated children was higher than the recommended age which shows a tendency to delay vaccinations. Further, a considerable number of children had an unknown vaccination status. Such delay in vaccination coverage together with the large group of children with an unknown vaccination status, might put Armenia at risk for future measles outbreaks.

## Data Availability

The DHS data sets including the data set used for this study are available upon requests made to MEASURE DHS (URL: https://www.dhsprogram.com/data/ available-datasets.cfm).

## Abbreviations

ADHS: Armenian Demographic Health Survey
MMR: Measles Mumps and Rubella vaccine
MCV: Measles containing vaccine
WHO: World Health Organization
OR: Odds Ratio
AOR: Adjusted Odds Ratio
CI: Confidence interval
SD: Standard deviation
